# A Novel Nomogram Model to Predict the Recurrence-Free Survival and Overall Survival of Hepatocellular Carcinoma

**DOI:** 10.3389/fonc.2022.946531

**Published:** 2022-07-22

**Authors:** Shu-Wen Zhang, Ning-Ning Zhang, Wen-Wen Zhu, Tian Liu, Jia-Yu Lv, Wen-Tao Jiang, Ya-Min Zhang, Tian-Qiang Song, Li Zhang, Yan Xie, Yong-He Zhou, Wei Lu

**Affiliations:** ^1^ Department of Hepatobiliary Oncology, Liver Cancer Center, National Clinical Research Center for Cancer, Key Laboratory of Cancer Prevention and Therapy, Tianjin’s Clinical Research Center for Cancer, Tianjin Medical University Cancer Institute and Hospital, Tianjin Medical University, Tianjin, China; ^2^ Department of Hepatology, Tianjin Third Central Hospital, Tianjin, China; ^3^ Department of Liver Transplantation, Tianjin First Center Hospital, NHC Key Laboratory for Critical Care Medicine, Key Laboratory of Transplantation, Chinese Academy of Medical Sciences, Tianjin, China; ^4^ Department of Hepatobiliary Surgery, Tianjin First Central Hospital, Tianjin Key Laboratory for Organ Transplantation, Tianjin, China; ^5^ Liver Cancer Center, National Clinical Research Center for Cancer, Key Laboratory of Cancer Prevention and Therapy, Tianjin’s Clinical Research Center for Cancer, Tianjin Medical University Cancer Institute and Hospital, Tianjin Medical University, Tianjin, China; ^6^ Tianjin Second People’s Hospital, Tianjin Medical Research Institute of Liver Disease, Tianjin, China

**Keywords:** hepatocellular carcinoma, Prognosis, nomogram, OS, RFS

## Abstract

**Background:**

Treatments for patients with early‐stage hepatocellular carcinoma (HCC) include liver transplantation (LT), liver resection (LR), radiofrequency ablation (RFA), and microwave ablation (MWA), are critical for their long-term survival. However, a computational model predicting treatment-independent prognosis of patients with HCC, such as overall survival (OS) and recurrence-free survival (RFS), is yet to be developed, to our best knowledge. The goal of this study is to identify prognostic factors associated with OS and RFS in patients with HCC and develop nomograms to predict them, respectively.

**Methods:**

We retrospectively retrieved 730 patients with HCC from three hospitals in China and followed them up for 3 and 5 years after invasive treatment. All enrolled patients were randomly divided into the training cohort and the validation cohort with a 7:3 ratio, respectively. Independent prognostic factors associated with OS and RFS were determined by the multivariate Cox regression analysis. Two nomogram prognostic models were built and evaluated by concordance index (C-index), calibration curves, area under the receiver operating characteristics (ROC) curve, time-dependent area under the ROC curve (AUC), the Kaplan–Meier survival curve, and decision curve analyses (DCAs), respectively.

**Results:**

Prognostic factors for OS and RFS were identified, and nomograms were successfully built. Calibration discrimination was good for both the OS and RFS nomogram prediction models (C-index: 0.750 and 0.746, respectively). For both nomograms, the AUC demonstrated outstanding predictive performance; the DCA shows that the model has good decision ability; and the calibration curve demonstrated strong predictive power. The nomograms successfully discriminated high-risk and low-risk patients with HCC associated with OS and RFS.

**Conclusions:**

We developed nomogram survival prediction models to predict the prognosis of HCC after invasive treatment with acceptable accuracies in both training and independent testing cohorts. The models may have clinical values in guiding the selection of clinical treatment strategies.

## Introduction

Liver cancer is one of the most prevalent and aggressive tumors, as well as the third leading cause of cancer-related mortality, with roughly 906,000 new cases and 83,0000 deaths reported in 2020 (1). Hepatocellular carcinoma (HCC) is the most common primary liver cancer comprising 75%–85% of all liver cancers. At present, there is no effective method for the treatment of advanced liver cancer, so the early treatment of liver cancer is very important for the prognosis of patients (2).

Early-stage HCC has been defined as Barcelona Clinic Liver Cancer (BCLC) 0 and A stages (BCLC 0/A), with curative therapy being the primary therapeutic option (2, 3). The treatment of early-stage HCC is feasible, but most patients with intermediate or advanced liver cancer have limited treatment opportunities and receive palliative treatment. The main curative treatments for early-stage HCC are liver resection (LR) and liver transplantation (LT). Radiofrequency ablation (RFA) or microwave ablation (MWA) is a viable minimally invasive therapy option for very early and early HCC, with equivalent outcomes to surgical resection (4–7). The high postoperative recurrence rate of liver cancer is the main obstacle affecting the low survival rate of patients with liver cancer (8–10).

There are several staging and grading systems for HCC, most notably the BCLC classification and the AJCC/TNM eight edition (11, 12). Several other factors were reported as potential predictor for the postoperative outcomes of patients with HCC, such as aspartate aminotransferase–to-platelet ratio index, albumin-bilirubin score (ALBI), the Model for Endstage Liver Disease (MELD) score and the Child-Pugh score (11, 13, 14). In addition, there are a few machine learning models for predicting the prognosis of cancer patients for other types of cancers based on histopathological images and multi-omics data (15–18). However, to our best knowledge, an accurate model predicting treatment-independent prognosis of patients with HCC, such as overall survival (OS) and recurrence-free survival (RFS), is yet to be developed. Although the treatment technology of HCC has made progress, the OS and RFS of patients with HCC are still relatively low. At present, there is still an urgent need for accurate models to predict OS and RFS in patients with HCC, guide individualized treatment, and prolong survival.

Nomogram is a user-friendly graphical prediction model tool that can help with therapeutic decision-making by quantifying the impacts of a variety of parameters (19). Therefore, we aimed to identify prognostic factors associated with OS and RFS in patients with HCC with different invasive treatments, including LT, LR, and minimally invasive approach (RFA or MWA) and develop nomograms to estimate 3-year and 5-year OS and RFS, respectively.

## Materials and methods

### Study Population

We retrospectively retrieved 730 patients with HCC underwent LT, LR, and RFA or MWA in three Chinese medical centers (Tianjin Medical University Cancer Institute and Hospital, Tianjin, China; The First Central Clinical School, Tianjin Medical University, Tianjin, China; and Clinical School of the Second People’s Hospital, Tianjin Medical University, Tianjin, China), and the follow-up deadline was on May 2019.

The inclusion criteria were as follows: (1) patients were validated by pathological diagnosis with primary HCC and assessed at BCLC 0/A; (2) patients underwent LT, LR, and RFA or MWA; (3) patients with complete clinic-pathological follow-up data; and (4) distant metastasis was not found.

### Data Collection and Follow-Ups

In our study, we collected the following clinical data from patients with HCC: (1) demographic characters, including age, gender, body mass index (BMI), and cirrhosis; (2) tumor size (largest tumor diameter), number, and location were estimated using magnetic resonance imaging (MRI) and/or computed tomography (CT) before treatment; (3) curative options, including LT, LR, and RFA or MWA; (4) microvascular invasion and differentiation grade were assessed postoperatively by postoperative pathology; and (5) BCLC classification was used to identify tumor stage. BMI was calculated using the following formula: BMI = weight (kg)/height (m^2^).

OS was defined as the time from the date of surgery to the date of death, and RFS was defined as the time from surgery to the date of first recurrence. All data were obtained from the first laboratory examination after admission. hepatitis C virus (HCV) and/or hepatitis B virus (HBV) infection as the presence of absence of anti-HCV or HBV surface antigen, respectively. Laboratory tests included routine blood tests, liver function tests, and alpha fetoprotein (AFP). Subgroup analysis will be performed based on Laboratory tests as follows: gamma-glutamyl transpeptidase (GGT) (<45 versus  ≥45, U/L), albumin (ALB) (<35 versus ≥35, g/L), prothrombin time (PT) (≤13 versus>13, s), aspartate aminotransferase (AST) (≤40 versus>40, U/L), total bilirubin (TBIL) (<20 versus ≥20, µmol/L), and AFP (<400 versus ≥400, ng/ml). MELD score grade, Child-Pugh Classification, and ALBI grade were also recorded. MELD score has been proved to be a predictor of survival in different end-stage liver diseases (20). Liver function was evaluated using Child-Pugh classification system. ALBI grade I: ≤−2.60 score; ALBI grade II: >−2.60 to ≤−1.39 score; ALBI grade III: >−1.39 score.

### Statistical Analysis

Statistical analysis was performed by R software (R Statistical Software, version 4.1.2). Independent prognostic factors were identified using multivariate Cox regression analyses. The results are presented as hazard ratio (HR) with 95% confidence intervals (CIs). Nomogram and calibration plots were constructed using R software. The C-index, ROC curve, calibration curve, and DCA were used to assess the nomogram. On the basis of the nomogram risk scores, Kaplan–Meier (K–M) survival curves were plotted for patients in the high-risk and low-risk groups.

## Results

### Patients’ Demographics and Clinical Characteristics

As shown in **Table 1**, a total of 730 patients diagnosed with primary HCC were included in our research. After using R software, patients were randomly allocated in a 7:3 ratio between the training cohort (512 patients) and the validation cohort (218 patients). In the OS analysis, the median follow-up period of the entire study cohort was 56.9 months (interquartile range, 2.8–116.3 months). In the RFS analysis, the median follow-up period for the overall research population was 41.3 months (interquartile range, 1.7–116.3 months). The training cohort was used to build the nomogram and internally validate the model, whereas the validation cohort was utilized for external verification. Both the training and the validation cohort had no statistical differences in their baseline characteristics in OS or RFS groups (**Table 1**).

**Table 1:** Demographic and clinical characteristics of patients.

Characters		Total patients(n = 730)	OS			RFS		
			Training Cohort (n = 512)	Validation Cohort(n = 218)	P- value	Training Cohort(n = 512)	Validation Cohort(n = 218)	P- value
Age (year)	<60/≥60	502/228	349/163	153/65	0.590	352/160	150/68	0.988
Gender	Female/Male	137/593	103/409	34/184	0.153	89/432	48/170	0.142
BMI (kg/m^2^)	<25/≥25	429/301	293/219	136/82	0.195	299/213	130/88	0.757
Cirrhosis	No/Yes	89/641	66/446	23/195	0.377	67/445	22/196	0.258
Tumor location	Left Lobe/Right Lobe/Both Lobe	127/571/32	90/396/26	37/175/6	0.690	84/405/23	43/166/9	0.301
Tumor Number	Solitary/Multiple	576/154	399/113	177/41	0.323	405/107	171/47	0.842
Tumor Size (cm)	≤3/3 < R ≤ 5	510/220	361/151	149/69	0.561	362/50	148/70	0.449
Operation	LT/LR/RFA or MWA	252/249/229	185/170/157	67/79/72	0.233	178/164/170	74/85/59	0.433
MELD score grade	<9/9-15/>15	459/218/53	319/154/39	140/64/14	0.577	319/156/37	140/62/16	0.663
Child-Pugh Classification	A/B	553/177	387/125	166/52	0.872	382/130	171/47	0.270
ALBI grade	I/II/III	344/344/42	236/249/27	108/95/15	0.564	240/240/32	104/104/10	0.689
**Laboratory examination**								
GGT (U/L)	<45/≥45	315/415	221/291	94/124	0.991	221/291	94/124	0.991
ALB (g/L)	<35/≥35	194/536	146/366	48/170	0.069	136/376	58/160	0.991
PT (S)	≤13/>13	347/383	243/269	104/114	0.952	246/266	101/117	0.671
AST (U/L)	≤40/>40	444/286	301/211	143/75	0.085	309/203	135/83	0.690
TBIL (µmol/L)	<20/≥20	412/318	267/225	125/93	0.749	288/224	124/94	0.875
AFP (ng/ml)	<400/≥400	632/98	443/69	189/29	0.950	440/72	192/26	0.439
HBV	No/Yes	136/594	100/412	36/182	0.338	95/417	41/177	0.936
HCV	No/Yes	670/60	466/46	204/14	0.249	468/44	202/16	0.573

BMI, body mass index; MELD, model for end-stage liver disease; ALBI, albumin-bilirubin; GGT, gamma-glutamyl transpeptidase; ALB, albumin; PT, prothrombin time; AST, aspartate aminotransferase; TBIL, total bilirubin; AFP, alpha fetoprotein; HBV, hepatitis B virus; HCV, hepatitis C virus.

In the entire cohort, 68.8% were aged < 60 years, 18.7% of the population were women, 58.8% were BMI < 25, and 87.8% of patients had cirrhosis. For all evaluated tumors, CT, MRI, or pathological examination results were available. Tumor size was defined as the largest diameter and 69.9% were less than 3 cm. There were 576 cases with a single tumor and 154 cases with multiple tumors. According to tumor location, 127 cases were in the left lobe, 571 cases were in the right lobe, and 32 cases were in both lobes. Pathological examination revealed microvascular invasion in 13.3% of all patients, whereas 55.3% were not. Child-Pugh grade and ALBI grade were used for assessment of hepatic function, and MELD score wasused to assess disease severity. After evaluation, most patients with liver cancer have good liver function.

The other components included GGT < 45 U/L (43.2% versus 56.8%), ALB < 35 g/L (26.6% versus 73.4%), PT ≤ 13 s (47.5% versus 52.5%), AST≤ 40 U/L (60.8% versus 39.2%), TBIL < 20 µmol/L (56.4% versus 43.6%), AFP < 400 ng/ml (86.6% versus 13.4%), HCV negative (18.6% versus 81.4%), and HBV negative (91.8% versus 8.2%). In the BCLC classification, grades 0/A accounted for 15.6% and 84.4%, respectively.

### Identification Prognostic Risk Factors For OS and RFS in 512 Patients With HCC

Multivariate analysis was conducted to identify the prognostic risk factors for OS and RFS. The results are shown in **Table 2**. In the OS analysis, gender (HR: 1.680; 95% CI: 1.002, 2.817; P = 0.049), BMI (HR: 1.621; 95% CI: 1.131, 2.324; P = 0.009), tumor number (HR: 2.165; 95% CI: 1.323, 3.544; P = 0.002), tumor size (HR: 2.180; 95% CI: 1.412, 3.391; P < 0.001), and operation (LR: HR: 3.905, 95% CI: 2.068, 7.372; P < 0.001; RFA or MWA: HR: 7.135, 95% CI: 3.906, 13.033; P < 0.001) were statistically significant differences. In the RFS analysis, tumor number (HR: 1.829; 95% CI: 1.223, 2.736; P = 0.003), operation (LR: HR: 6.019; 95% CI: 3.588, 10.098; P < 0.001; RFA or MWA: HR: 12.089; 95% CI: 7.417, 19.703; P < 0.001), GGT (HR: 1.650; 95% CI 1.199, 2.269; P = 0.002), and HCV (HR: 0.430; 95% CI: 0.213, 0.870; P = 0.019) were considered statistically different.

**Table 2:** Multivariate analyses for OS and RFS in patients with 512 HCC (training cohort).

Characters		OS		RFS	
		HR (95% CI)	p-value	HR (95% CI)	p-value
Age (year)	<60/≥60	1.040 (0.696–1.555)	0.848	0.900 (0.666–1.216)	0.491
Gender	Female/Male	1.680 (1.002–2.817)	0.049*	1.233 (0.837–1.814)	0.287
BMI (kg/m^2^)	<25/≥25	1.621 (1.131–2.324)	0.009*	1.272 (0.959–1.686)	0.095
Cirrhosis	No/Yes	0.705 (0.393–1.265)	0.241	0.954 (0.613–1.483)	0.833
Tumor location	Left Lobe	Reference		Reference	
	Right Lobe	0.967 (0.598–1.565)	0.893	0.859 (0.588–1.253)	0.429
	Both Lobe	0.750 (0.243–2.315)	0.617	1.271 (0.568–2.845)	0.559
Tumor Number	Solitary/Multiple	2.165 (1.323–3.544)	0.002*	1.829 (1.223–2.736)	0.003*
Tumor Size (cm)	≤3/3 < R ≤ 5	2.180 (1.412–3.391)	<0.001*	1.370 (0.984–1.907)	0.062
Operation	LT	Reference		Reference	
	LR	3.905 (2.068–7.372)	<0.001*	6.019 (3.588–10.098)	<0.001*
	RFA or MWA	7.135 (3.906–13.033)	<0.001*	12.089 (7.417–19.703)	<0.001*
MELD score grade	<9	Reference		Reference	
	5–9	0.997 (0.552–1.799)	0.991	1.220 (0.802–1.870)	0.348
	>15	0.843 (0.291–2.444)	0.753	1.286 (0.572–2.895)	0.543
Child–Pugh Classification	A/B	1.090 (0.585–2.032)	0.786	0.973 (0.599–1.582)	0.913
ALBI grade	I	Reference		Reference	
	II	0.898 (0.543–1.486)	0.676	1.141 (0.787–1.655)	0.486
	III	1.249 (0.485–3.215)	0.645	1.443 (0.648–3.214)	0.369
**Laboratory examination**					
GGT (U/L)	<45/≥45	1.396 (0.934–2.088)	0.104	1.650 (1.199–2.269)	0.002*
ALB (g/L)	<35/≥35	0.828 (0.461–1.487)	0.527	0.971 (0.621–1.519)	0.900
PT (S)	≤13/>13	1.191 (0.722–1.964)	0.495	0.885 (0.604–1.297)	0.531
AST (U/L)	≤40/>40	1.448 (0.885–2.367)	0.140	0.968 (0.678–1.381)	0.857
TBIL (µmol/L)	<20/≥20	0.919 (0.556–1.519)	0.741	0.823 (0.565–1.198)	0.309
AFP (ng/ml)	<400/≥400	1.159 (0.682–1.971)	0.586	1.449 (0.963–2.179)	0.075
HBV	No/Yes	0.737 (0.445–1.220)	0.235	0.741 (0.481–1.142)	0.175
HCV	No/Yes	0.511 (0.223–1.170)	0.112	0.430 (0.213–0.870)	0.019*

*OS, overall survival; RFS, recurrence-free survival; HCC, hepatocellular carcinoma; BMI, body mass index; MELD, model for end-stage liver disease; ALBI, albumin-bilirubin; GGT, gamma-glutamyl transpeptidase; ALB, albumin; PT, prothrombin time; AST, aspartate aminotransferase; TBIL, total bilirubin; AFP, alpha fetoprotein; HBV, hepatitis B virus; HCV, hepatitis C virus.

### Nomogram for OS and RFS Construction and Performance Evaluation

**Figure 1:**
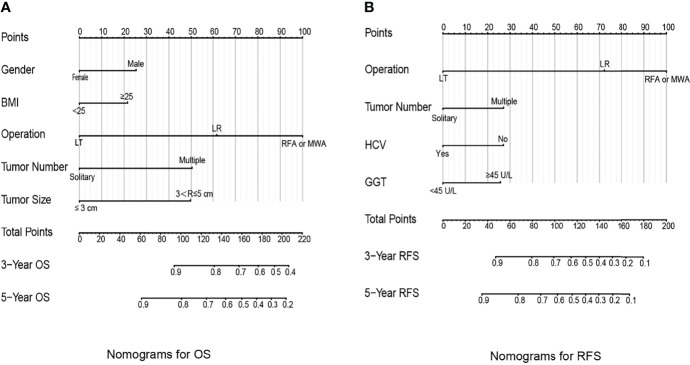
Prognostic nomograms to predict the overall survival **(A)** and recurrence-free survival **(B)** of hepatocellular carcinoma patients.

**Figure 2:**
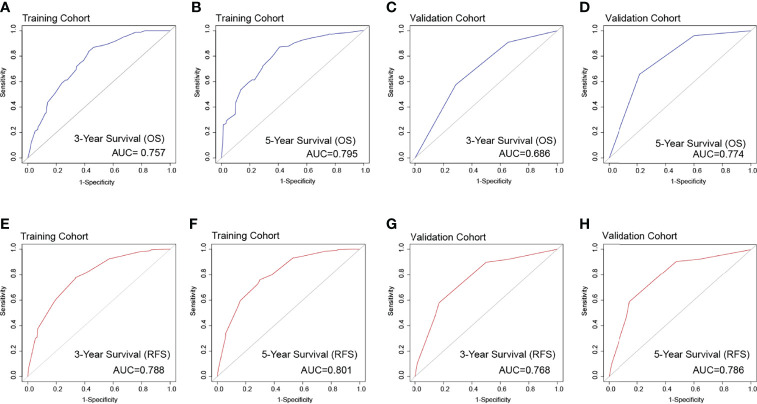
The overall survival ROC curves for 3 years **(A)** and 5 years **(B)**, respectively, validated by the model establishment training cohort; ROC curves for 3 years **(C)** and 5 years **(D)**, respectively, validated by the validation group. The recurrence-free survival ROC curves in the training cohort [**(E)** 3 years; **(F)** 5 years] and the validation cohort [**(G)** 3 years; **(H)** 5 years].

**Figure 3:**
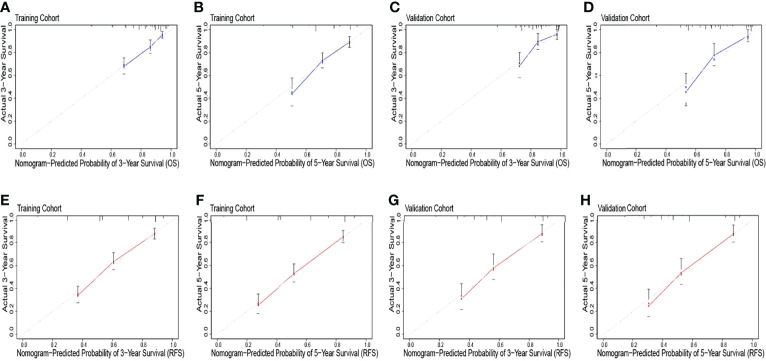
The overall survival calibration curve for predicting patient survival at 3 years **(A)** and 5 years **(B)** in the training cohort and 3 years **(C)** and 5 years **(D)** in the validation cohort. The recurrence-free survival calibration curve for predicting patient survival at 3 years **(E)** and 5 years **(F)** in the training cohort and 3 years **(G)** and 5 years **(H)** in the validation cohort.

**Figure 4:**
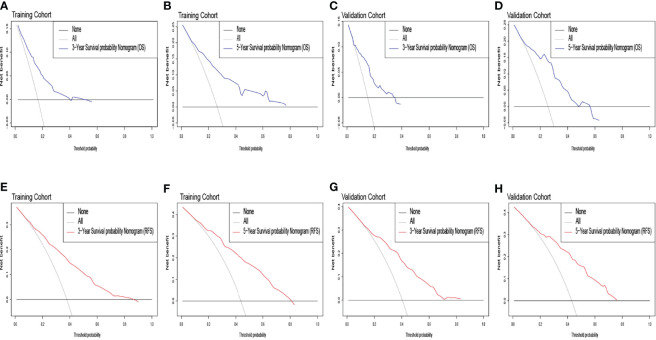
The overall survival decision curve in the training cohort [**(A)** 3 years; **(B)** 5 years] and the validation cohort [**(C)** 3 years; **(D)** 5 years]. The recurrence-free survival decision curve in the training cohort [**(E)** 3 years; **(F)** 5 years] and the validation cohort [**(G)** 3 years; **(H)** 5 years].

The identification prognostic risk factors of OS and RFS were included to create prognostic nomograms to assess the 3-year and 5-year OS and RFS of patients with HCC (**Figure 1**). Nomograms predicted 3-year and 5-year OS and RFS indicated that operation factors had major impacts on patient prognosis. The 3-year and 5-year AUCs for OS were 0.757 and 0.795, respectively, and were 0.788 and 0.801 for RFS, respectively (**Figures 2A, B, E, F**). In the OS testing cohort, the AUCs of 3-year and 5-year AUCs were 0.686 and 0.774, respectively (**Figures 2C, D**). In the RFS testing cohort, the AUCs of 3-year and 5-year AUCs were 0.768 and 0.786, respectively (**Figures 2G, H**). On the 3-year and 5-year calibration plots of OS and RFS, calibration curves revealed the consistency of the nomogram between predicted and actual observed and showed that the nomograms were highly consistent in both training and validation cohorts (**Figure 3**). In addition, the DCA curves revealed that the nomogram had a high prediction efficiency for CSS of patients with HCC in both OS and RFS (**Figure 4**).

**Figure 5:**
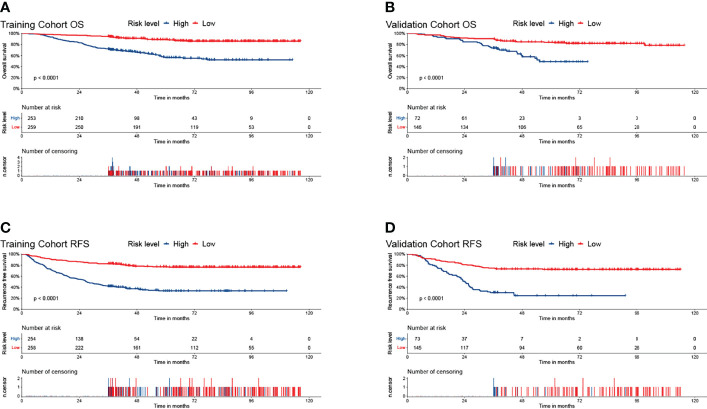
The Kaplan–Meier survival curves for low- and high- risk groups in patients with HCC based on risk scores. The overall survival Kaplan–Meier survival curves in the training cohort **(A)** and the validation cohort **(B)**. The recurrence-free survival Kaplan–Meier survival curves in the training cohort **(C)** and the validation cohort **(D)**.

Furthermore, the K–M survival curve revealed that high-risk individuals have a worse prognosis than low-risk patients (**Figure 5**). In both the OS and RFS analyses, the nomogram models outperformed the other factors, with C-indices of 0.750 (OS, 95% CI: 0.713–0.787) and 0.746 (RFS, 95% CI: 0.715–0.777) in the training cohort and 0.794 (OS, 95% CI: 0.739–0.849) and 0.757 (RFS, 95% CI: 0.708–0.806) in the validation cohort (**Table 3**).

**Table 3:** Ranking of clinical staging system using C-index for OS and RFS in the training and validation cohorts.

Variables		Training Cohort		Validation Cohort	
		c-index	95% CI	c-index	95% CI
**OS**	Nomogram	0.750	0.713–0.787	0.794	0.739–0.849
	Operation	0.648	0.605–0.691	0.685	0.624–0.746
	Tumor Size	0.571	0.528–0.614	0.508	0.443–0.573
	BMI	0.550	0.507–0.593	0.544	0.483–0.605
	Tumor Number	0.530	0.491–0.569	0.511	0.454–0.568
	Gender	0.526	0.493–0.559	0.524	0.481–0.567
**RFS**	Nomogram	0.746	0.715–0.777	0.757	0.708–0.806
	Operation	0.684	0.653–0.715	0.678	0.625–0.731
	GGT	0.540	0.507–0.573	0.525	0.474–0.576
	HCV	0.520	0.502–0.538	0.506	0.477–0.535
	Tumor Number	0.512	0.483–0.541	0.513	0.470–0.556

OS, overall survival; RFS, recurrence-free survival; BMI, body mass index; GGT, gamma-glutamyl transpeptidase; HCV, hepatitis C virus.

## Discussion

To our knowledge, this is the first attempt to construct prognostic nomograms for OS and RFS to predict the prognosis of patients with HCC with different invasive treatments, including LT, LR, and minimally invasive approach (RFA or MWA). Although our nomograms had good performance in predicting survival at 3 and 5 years in patients with HCC, it might be further improved by integrating more types of data, such as pathological images and multi-omics data (17, 21), and by applying more advanced classification algorithms as used in cancer diagnosis and other biological problems (15, 22). In the future, we will explore these directions.

Multivariate analyses revealed that the choice of different invasive treatments (including LT, LR, RFA, and MWA) may be an important independent prognostic factor in the patients with HCC. We have further shown that minimally invasive approach (RFA or MWA) was the strongest predictor, followed by LR and LT, but some studies reported retrospective studies contrary to our study. Resection is the preferred option for patients with early-stage liver cancer (BCLC 0/A) and confer 5-year OS rates of 64.2% (4, 23, 24). Studies by other investigators suggest that LT is the best treatment option for patients with early HCC (25, 26). Minimally invasive surgery has undeniably played a significant role in HCC treatment in recent years. RFA or MWA was a common type of minimally invasive surgery, they showed comparable outcome and similar survival rates with LR (27–29). Compared with our previous nomogram studies, we were fortunate to have access to treatment-independent prognostic factors, including LT, LR, RFA, and MWA (30). As a result, we created predictive nomograms to predict OS and RFS in patients with HCC undergone various invasive therapies. The nomograms were validated as an effective tool for predicting long-term outcomes. The current findings will need to be confirmed by larger prospective investigations into why different invasive treatments have different outcomes in the future. However, it is worthy noticing that we only considered single factors in the current analyses and ignore the relationship among these factors, for example, correlation and collinearity. Theoretically, integrating the correlation (collinearity) among the factors into multivariate analyses will increase the size of feature space and should increase the performance of our model. However, it will also make the model more complicated, and thus, we consider it as a future work.

In our study, tumor number (solitary versus multiple) was shown to play a role in the OS nomogram as well as in the RFS nomogram. Several studies have identified the presence of multiple tumors as a crucial risk factor for recurrence, which is consistent with the findings of our study (31, 32). Patients with HCC have a poor prognosis due to metastasis and recurrence. There is a strong association between tumor number > 1 and 3-year and 5-year OS, according to Xiao et al. (33). Compared with single tumor, multiple tumors are more prone to microvascular invasion (MVI) which will lead to increased tumor recurrence after surgery (34, 35).

In the OS nomogram, gender, BMI, and tumor size were also independent prognostic risk factors of patients with HCC. Gender was a prognostic factor also find in the nomogram for predicting the prognosis of patients with HCC with pulmonary metastases (36). In Global Cancer Statistics 2020, the incidence and mortality rates of liver cancer are two to three times greater in men than in women (1). Women are generally at lower risk for the development of HCC compared with men, and this may be due, in part, to the beneficial effects of sex hormones (37, 38). Sex hormone therapy is one of the potential development avenues of HCC treatment as part of multimodal liver cancer treatment.

In patients undergoing LR for HCC, preoperative bodyweight is linked to long-term prognosis (39). Furthermore, BMI ≥ 25 kg/m^2^ negatively affected the surgical outcomes of patients with HBV-related HCC (BMI < 25 kg/m^2^ group: 3-, 5-, and 8-year survival rates of 88.3%, 81.6%, and 73.9%, respectively, versus BMI ≥ 25 kg/m^2^ group 85.8%, 61.0%, and 48.1%, respectively) (40). Previous study had revealed that the beneficial BMI level for patients with HCC following MWA is 21.5 to 23.1 kg/m^2^ and can therefore achieve a longer survival time (41). Hence, it is critical for patients with HCC with weight concerns to confirm the beneficial BMI levels and the need for further research for different treatments.

Tumor size was not associated with RFS, and increasing trends toward the mortality of all patients with OS were observed for patients with a tumor size of ≤3 cm (21.9%) compared with patients with a tumor size of 3< R ≤ 5 cm (33.2%), which was consistent with previous work. However, in a large international study, large tumor size was the key parameter related to early HCC recurrence after LR, and they built a preoperative model for RFS in the entire cohort (low risk: 2-year RFS 64.8%; intermediate risk: 2-year RFS 42.5%; and high risk: 2-year RFS 20.7%) (42). A previous study has also reported that tumor size was not an independent prognostic factor of OS or RFS after curative resection and did not influence survival in patients with HCC without vascular invasion (43). We pointed out that tumor size is an important risk factor for OS and RFS, but different invasive treatments can obtain good clinical results and effectively reduce the recurrence rate. Therefore, tumor size is not a prognostic risk factor for the RFS nomogram.

In the RFS nomogram, there were prognostic risk factors also include GGT and HCV. A 384-patient study has shown that GGT > 50 U/L and indocyanine green retention of 15 min (ICG-R15) > 10% were identified as preoperative independent risk factors affecting 1-, 3-, and 5-year RFS (72.8%, 43.3%, and 27%, respectively) (44). A meta-analysis shows that high pretreatment serum GGT level is significantly correlated with poor survival and unfavorable clinicopathological features in patients with HCC, suggesting that pretreatment serum GGT may be an economical and effective prognostic biomarker for patients with HCC (45). Surgical patients who received HCV treatment had improved RFS compared with those who did not (91 vs. 80 months, p = 0.03) (46). Our findings are consistent with the few prior studies that found patients with HCC with HCV infection as a protective prognostic factor for patients with HCC in different invasive approaches to treatments. Patients with non-viral HCC have poorer prognosis than those with HCV-HCC (47). The reason why patients with HCV-HCC improve survival is that, possibly, antiviral therapy and virus replication reduced cancerous HCV‐HCC tissues (46, 48–50). As a result, more research studies into the impact of antiviral medication on the outcomes of patients with HCC following surgery are needed.

## Conclusions

Our study identified prognostic risk factors for OS and RFS in patients with early-stage HCC treated with different invasive treatments (including LT, LR, RFA, and MWA), and we established and validation two prognostic nomograms. Two nomograms will be clinical settings for customized risk assessment and surgical decision-making. Furthermore, developing personalized treatment regimens for patients with different prognoses is beneficial.

## Data Availability Statement

The original contributions presented in the study are included in the article/supplementary material, further inquiries can be directed to the corresponding author.

## Ethics Statement

The studies involving human participants were reviewed and approved by the Ethics Reviewer Board of Tianjin Medical University Cancer Institute and Hospital (bc2019082). The patients/participants provided their written informed consent to participate in this study.

## Author Contributions

Study concept: WL and N-NZ. Study design: WL, N-NZ, S-WZ, and W-WZ. Data collection: S-WZ, W-WZ, J-YL, W-TJ, Y-MZ, T-QS, LZ, YX, and Y-HZ. Analysis and interpretation of data: S-WZ, TL, and W-WZ. Manuscript drafting: S-WZ, W-WZ, TL, and J-YL. Revising the manuscript: N-NZ, and WL. Acquisition and review of data, provision of critical comments or suggestions: all authors. Manuscript final version approval: all authors.

## Conflict of Interest

The authors declare that the research was conducted in the absence of any commercial or financial relationships that could be construed as a potential conflict of interest.

## Publisher’s Note

All claims expressed in this article are solely those of the authors and do not necessarily represent those of their affiliated organizations, or those of the publisher, the editors and the reviewers. Any product that may be evaluated in this article, or claim that may be made by its manufacturer, is not guaranteed or endorsed by the publisher.

## References

[1] Sung H Ferlay J Siegel RL Laversanne M Soerjomataram I Jemal A. Global Cancer Statistics 2020: GLOBOCAN Estimates of Incidence and Mortality Worldwide for 36 Cancers in 185 Countries. CA Cancer J Clin (2021) 71:209–49. doi: 10.3322/caac.21660 33538338

[2] Bruix J Reig M Sherman M. Evidence-Based Diagnosis, Staging, and Treatment of Patients With Hepatocellular Carcinoma. Gastroenterology (2016) 150:835–53. doi: 10.1053/j.gastro.2015.12.041 26795574

[3] Tsilimigras DI Bagante F Sahara K Moris D Hyer JM Wu L. Prognosis After Resection of Barcelona Clinic Liver Cancer (BCLC) Stage 0, A, and B Hepatocellular Carcinoma: A Comprehensive Assessment of the Current BCLC Classification. Ann Surg Oncol (2019) 26:3693–700. doi: 10.1245/s10434-019-07580-9 31267302

[4] Morise Z Kawabe N Tomishige H Nagata H Kawase J Arakawa S. Recent Advances in the Surgical Treatment of Hepatocellular Carcinoma. World J Gastroenterol (2014) 20:14381–92. doi: 10.3748/wjg.v20.i39.14381 PMC4202367 25339825

[5] Di Sandro S Benuzzi L Lauterio A Botta F De Carlis R Najjar M. Single Hepatocellular Carcinoma Approached by Curative-Intent Treatment: A Propensity Score Analysis Comparing Radiofrequency Ablation and Liver Resection. Eur J Surg Oncol (2019) 45:1691–9. doi: 10.1016/j.ejso.2019.04.023 31072620

[6] Gupta P Maralakunte M Kumar MP Chandel K Chaluvashetty SB Bhujade H. Overall Survival and Local Recurrence Following RFA, MWA, and Cryoablation of Very Early and Early HCC: A Systematic Review and Bayesian Network Meta-Analysis. Eur Radiol (2021) 31:5400–8. doi: 10.1007/s00330-020-07610-1 33439319

[7] Yang Y Yu H Tan X You Y Liu F Zhao T. Liver Resection Versus Radiofrequency Ablation for Recurrent Hepatocellular Carcinoma: A Systematic Review and Meta-Analysis. Int J Hyperthermia (2021) 38:875–86. doi: 10.1080/02656736.2021.1933218 34078221

[8] Tabrizian P Jibara G Shrager B Schwartz M Roayaie S. Recurrence of Hepatocellular Cancer After Resection: Patterns, Treatments, and Prognosis. Ann Surg (2015) 261:947–55. doi: 10.1097/SLA.0000000000000710 25010665

[9] Liu H Qiu C Wang B Bing P Tian G Zhang X. Evaluating DNA Methylation, Gene Expression, Somatic Mutation, and Their Combinations in Inferring Tumor Tissue-Of-Origin. Front Cell Dev Biol (2021) 9:619330. doi: 10.3389/fcell.2021.619330 34012960 PMC8126648

[10] Ivanics T Murillo Perez CF Claasen M Patel MS Morgenshtern G Erdman L. Dynamic Risk Profiling of HCC Recurrence After Curative Intent Liver Resection. Hepatology (2022). doi: 10.1002/hep.32411 35178739

[11] Liao X Zhang D. The 8th Edition American Joint Committee on Cancer Staging for Hepato-Pancreato-Biliary Cancer: A Review and Update. Arch Pathol Lab Med (2021) 145:543–53. doi: 10.5858/arpa.2020-0032-RA 32223559

[12] Reig M Forner A Rimola J Ferrer-Fabrega J Burrel M Garcia-Criado A. BCLC Strategy for Prognosis Prediction and Treatment Recommendation: The 2022 Update. J Hepatol (2022) 76:681–93. doi: 10.1016/j.jhep.2021.11.018 PMC8866082 34801630

[13] Kim KM Shim SG Sinn DH Song JE Kim BS Kim HG. Child-Pugh, MELD, MELD-Na, and ALBI Scores: Which Liver Function Models Best Predicts Prognosis for HCC Patient With Ascites? Scand J Gastroenterol (2020) 55:951–7. doi: 10.1080/00365521.2020.1788139 32698637

[14] Shi JY Sun LY Quan B Xing H Li C Liang L. A Novel Online Calculator Based on Noninvasive Markers (ALBI and APRI) for Predicting Post-Hepatectomy Liver Failure in Patients With Hepatocellular Carcinoma. Clin Res Hepatol Gastroenterol (2021) 45:101534. doi: 10.1016/j.clinre.2020.09.001 33067168

[15] Song Z Chen X Shi Y Huang R Wang W Zhu K. Evaluating the Potential of T Cell Receptor Repertoires in Predicting the Prognosis of Resectable Non-Small Cell Lung Cancers. Mol Ther Methods Clin Dev (2020) 18:73–83. doi: 10.1016/j.omtm.2020.05.020 32995352 PMC7488751

[16] Yang J Ju J Guo L Ji B Shi S Yang Z. Prediction of HER2-Positive Breast Cancer Recurrence and Metastasis Risk From Histopathological Images and Clinical Information *via* Multimodal Deep Learning. Comput Struct Biotechnol J (2022) 20:333–42. doi: 10.1016/j.csbj.2021.12.028 PMC8733169 35035786

[17] Yang M Yang H Ji L Hu X Tian G Wang B. A Multi-Omics Machine Learning Framework in Predicting the Survival of Colorectal Cancer Patients. Comput Biol Med (2022) 146:105516. doi: 10.1016/j.compbiomed.2022.105516 35468406

[18] Ye Z Zhang Y Liang Y Lang J Zhang X Zang G. Cervical Cancer Metastasis and Recurrence Risk Prediction Based on Deep Convolutional Neural Network. Curr Bioinf (2022) 17:164–73. doi: 10.2174/1574893616666210708143556

[19] Iasonos A Schrag D Raj GV Panageas KS. How to Build and Interpret a Nomogram for Cancer Prognosis. J Clin Oncol (2008) 26:1364–70. doi: 10.1200/JCO.2007.12.9791 18323559

[20] Kamath PS Wiesner RH Malinchoc M Kremers W Therneau TM Kosberg CL. A Model to Predict Survival in Patients With End-Stage Liver Disease. Hepatology (2001) 33:464–70. doi: 10.1053/jhep.2001.22172 11172350

[21] Liu X Yuan P Li R Zhang D An J Ju J. Predicting Breast Cancer Recurrence and Metastasis Risk by Integrating Color and Texture Features of Histopathological Images and Machine Learning Technologies. Comput Biol Med (2022) 146:105569. doi: 10.1016/j.compbiomed.2022.105569 35751195

[22] Meng Y Lu C Jin M Xu J Zeng X Yang J. A Weighted Bilinear Neural Collaborative Filtering Approach for Drug Repositioning. Brief Bioinform (2022) 23(2): bbab581. doi: 10.1093/bib/bbab581 35039838

[23] Sapisochin G Castells L Dopazo C Bilbao I Minguez B Lazaro JL. Single HCC in Cirrhotic Patients: Liver Resection or Liver Transplantation? Long-Term Outcome According to an Intention-to-Treat Basis. Ann Surg Oncol (2013) 20:1194–202. doi: 10.1245/s10434-012-2655-1 22965574

[24] Tsilimigras DI Mehta R Moris D Sahara K Bagante F Paredes AZ. Utilizing Machine Learning for Pre- and Postoperative Assessment of Patients Undergoing Resection for BCLC-0, A and B Hepatocellular Carcinoma: Implications for Resection Beyond the BCLC Guidelines. Ann Surg Oncol (2020) 27:866–74. doi: 10.1245/s10434-019-08025-z 31696396

[25] Melloul E Lesurtel M Carr BI Clavien PA. Developments in Liver Transplantation for Hepatocellular Carcinoma. Semin Oncol (2012) 39:510–21. doi: 10.1053/j.seminoncol.2012.05.008 22846868

[26] Ziogas IA Ye F Zhao Z Matsuoka LK Montenovo MI Izzy M. Population-Based Analysis of Hepatocellular Carcinoma in Children: Identifying Optimal Surgical Treatment. J Am Coll Surg (2020) 230:1035–1044.e1033. doi: 10.1016/j.jamcollsurg.2020.03.024 32272204

[27] Ahn KS Kang KJ. Appropriate Treatment Modality for Solitary Small Hepatocellular Carcinoma: Radiofrequency Ablation vs. Resection vs. Transplantation? Clin Mol Hepatol (2019) 25:354–9. doi: 10.3350/cmh.2018.0096 PMC6933127 31006225

[28] Gui CH Baey S D'cruz R T Shelat VG. Trans-Arterial Chemoembolization + Radiofrequency Ablation Versus Surgical Resection in Hepatocellular Carcinoma - A Meta-Analysis. Eur J Surg Oncol (2020) 46:763–71. doi: 10.1016/j.ejso.2020.01.004 31937433

[29] Ricci AD Rizzo A Bonucci C Tavolari S Palloni A Frega G. The (Eternal) Debate on Microwave Ablation Versus Radiofrequency Ablation in BCLC-A Hepatocellular Carcinoma. In Vivo (2020) 34:3421–9. doi: 10.21873/invivo.12181 PMC7811655 33144450

[30] Zhang N Jiang W Zhang Y Song TQ Lv J Gu J. Individualised Tailored Assessment of Therapeutic Alternatives for HCC Patients Within the Milan Criteria. Gut (2020) 69:1893–5. doi: 10.1136/gutjnl-2019-320073 PMC7497580 31748202

[31] Lei Z Li J Wu D Xia Y Wang Q Si A. Nomogram for Preoperative Estimation of Microvascular Invasion Risk in Hepatitis B Virus-Related Hepatocellular Carcinoma Within the Milan Criteria. JAMA Surg (2016) 151:356–63. doi: 10.1001/jamasurg.2015.4257 26579636

[32] Xu XF Xing H Han J Li ZL Lau WY Zhou YH. Risk Factors, Patterns, and Outcomes of Late Recurrence After Liver Resection for Hepatocellular Carcinoma: A Multicenter Study From China. JAMA Surg (2019) 154:209–17. doi: 10.1001/jamasurg.2018.4334 PMC6439634 30422241

[33] Xiao YB Zhang B Wu YL. Radiofrequency Ablation Versus Hepatic Resection for Breast Cancer Liver Metastasis: A Systematic Review and Meta-Analysis. J Zhejiang Univ Sci B (2018) 19:829–43. doi: 10.1631/jzus.B1700516 PMC6238116 30387333

[34] Pawlik TM Delman KA Vauthey JN Nagorney DM Ng IO Ikai I. Tumor Size Predicts Vascular Invasion and Histologic Grade: Implications for Selection of Surgical Treatment for Hepatocellular Carcinoma. Liver Transpl (2005) 11:1086–92. doi: 10.1002/lt.20472 16123959

[35] Lee S Kang TW Song KD Lee MW Rhim H Lim HK. Effect of Microvascular Invasion Risk on Early Recurrence of Hepatocellular Carcinoma After Surgery and Radiofrequency Ablation. Ann Surg (2021) 273:564–71. doi: 10.1097/SLA.0000000000003268 31058694

[36] Zhou Y Zhou X Ma J Zhang W Yan Z Luo J. Nomogram for Predicting the Prognosis of Patients With Hepatocellular Carcinoma Presenting With Pulmonary Metastasis. Cancer Manag Res (2021) 13:2083–94. doi: 10.2147/CMAR.S296020 PMC7935331 33688251

[37] Giannitrapani L Soresi M La Spada E Cervello M D'alessandro N Montalto G. Sex Hormones and Risk of Liver Tumor. Ann N Y Acad Sci (2006) 1089:228–36. doi: 10.1196/annals.1386.044 17261770

[38] Shen M Xu M Zhong F Crist MC Prior AB Yang K. A Multi-Omics Study Revealing the Metabolic Effects of Estrogen in Liver Cancer Cells Hepg2. Cells (2021) 10(2):455. doi: 10.3390/cells10020455 33672651 PMC7924215

[39] Yu JJ Shen F Chen TH Liang L Han J Xing H. Multicentre Study of the Prognostic Impact of Preoperative Bodyweight on Long-Term Prognosis of Hepatocellular Carcinoma. Br J Surg (2019) 106:276–85. doi: 10.1002/bjs.10981 30199100

[40] Hashimoto M Tashiro H Kobayashi T Kuroda S Hamaoka M Ohdan H. Influence of Higher BMI for Hepatitis B- and C-Related Hepatocellular Carcinomas. Langenbecks Arch Surg (2017) 402:745–55. doi: 10.1007/s00423-017-1589-2 28534136

[41] Dou JP Han ZY Liu F Cheng Z Yu X Yu J. Beneficial Body Mass Index to Enhance Survival Outcomes in Patients With Early-Stage Hepatocellular Carcinoma Following Microwave Ablation Treatment. Int J Hyperthermia (2020) 37:110–8. doi: 10.1080/02656736.2020.1712482 31969030

[42] Chan AWH Zhong J Berhane S Toyoda H Cucchetti A Shi K. Development of Pre and Post-Operative Models to Predict Early Recurrence of Hepatocellular Carcinoma After Surgical Resection. J Hepatol (2018) 69:1284–93. doi: 10.1016/j.jhep.2018.08.027 30236834

[43] Zhang H Yuan SX Dai SY Zhang JM Huang X Lu CD. Tumor Size Does Not Independently Affect Long-Term Survival After Curative Resection of Solitary Hepatocellular Carcinoma Without Macroscopic Vascular Invasion. World J Surg (2014) 38:947–57. doi: 10.1007/s00268-013-2365-2 24258262

[44] Song P Inagaki Y Wang Z Hasegawa K Sakamoto Y Arita J. High Levels of Gamma-Glutamyl Transferase and Indocyanine Green Retention Rate at 15 Min as Preoperative Predictors of Tumor Recurrence in Patients With Hepatocellular Carcinoma. Med (Baltimore) (2015) 94:e810. doi: 10.1097/MD.0000000000000810 PMC4616400 26020384

[45] Sun P Li Y Chang L Tian X. Prognostic and Clinicopathological Significance of Gamma-Glutamyltransferase in Patients With Hepatocellular Carcinoma: A PRISMA-Compliant Meta-Analysis. Med (Baltimore) (2019) 98:e15603. doi: 10.1097/MD.0000000000015603 PMC6531078 31083251

[46] Turgeon MK Lee RM Gamboa AC Yopp A Ryon EL Goel N. Impact of Hepatitis C Treatment on Long-Term Outcomes for Patients With Hepatocellular Carcinoma: A United States Safety Net Collaborative Study. HPB (Oxford) (2021) 23:422–33. doi: 10.1016/j.hpb.2020.07.012 PMC7970452 32778389

[47] Noda Y Kawaguchi T Kuromatsu R Komukai S Nakano M Niizeki T. Prognostic Profile of Patients With non-Viral Hepatocellular Carcinoma: A Comparative Study With Hepatitis C Virus-Related Hepatocellular Carcinoma Using Data Mining Analysis. Oncol Lett (2019) 18:227–36. doi: 10.3892/ol.2019.10285 PMC6540186 31289492

[48] Shindoh J Hasegawa K Takemura N Omichi K Ishizawa T Aoki T. Hepatitis C Viral Load Predicts Tumor Recurrence After Curative Resection of Hepatocellular Carcinoma Regardless of the Genotype of Hepatitis C Virus. Hepatol Int (2014) 8:112–20. doi: 10.1007/s12072-013-9507-3 26202412

[49] Harouaka D Engle RE Wollenberg K Diaz G Tice AB Zamboni F. Diminished Viral Replication and Compartmentalization of Hepatitis C Virus in Hepatocellular Carcinoma Tissue. Proc Natl Acad Sci USA (2016) 113:1375–80. doi: 10.1073/pnas.1516879113 PMC4747736 26787866

[50] Manthravadi S Paleti S Pandya P. Impact of Sustained Viral Response Postcurative Therapy of Hepatitis C-Related Hepatocellular Carcinoma: A Systematic Review and Meta-Analysis. Int J Cancer (2017) 140:1042–9. doi: 10.1002/ijc.30521 27861842

